# Mathematical Measurement

**Published:** 1878-06

**Authors:** 


"THE MATHEMATICAL MEASUREMENT OF THE LUNGS AS A SIGN OF CONSUMPTION.

"The lungs contain air; and their object is to receive, hold, and expel air; a certain amount of this air is necessary to the health of any individual, but that amount must vary in proportion to the size and age of a person, as much as the healthful amount of blood is proportionate to the size and age.

"It is known how much air a man's lungs, in perfect and full healthful operation, should hold, by measuring it as we would measure water, by transferring it from a vessel whose capacity was not known into one whose capacity was known. If, then, I find that every man of thousands, who is in perfect health, emits a certain amount of air from his lungs, I conclude that any other man, *under similar circumstances*, who gives from his lungs an equal amount of air, must be in good health, as far as his lungs are concerned, and every year accumulates its additional proofs of the same great fact, and when it is known that the lungs work *fully* and well, an immense burthen is at once removed from the mind of the physician, as well as patient, for he has less to do—the patient has less to dread.

"All that the *Spirometer* does, (or *Breath-Measurer*, which is its literal signification,) is to measure the amount of air contained in any man's lungs with mathematical certainty and precision, down to the fraction of a single cubic inch. Thus far the patient can see, as well as the physician, what is his actual measure; and by comparing it with what it ought to be in health, he can have some idea of what he has to do, and of his present condition.

"We all must know that if a man's lungs in health should hold three hundred cubic inches, they would, if half gone, certainly not measure over one hundred and fifty, *and so of any other proportion*, down to an inch.

"The two important uses to be made of this most invaluable principal are—

*First.* If a man can only expire his full healthful quota of air, he most assuredly cannot have actual consumption, whatever else may be the matter with him, and the knowledge of this one fact alone, arrived at by such unmistakable evidence, is of incomputable worth to any invalid, not only relieving him of the weight of a million mill-stones, but in affording him an important means of restoration—*hopefulness*, for we almost all instinctively feel, if it is not consumption there is at least a chance of life; but if it is consumption there is no hope.

"*Second.* The next important practical deduction is of a two-fold character.

"If the lungs do not give out their full healthful amount of air, it is because they are actually affected or are threatened. The instrument does not tell this, it must be determined by the mature judgment of the experienced physician.

"If the lungs be in a consumptive decay, the pulse and auscultation, with the data already afforded by measurement, will detect this state of things, with a degree of certainty which is most admirable; and this certainty is made doubly sure, if being under treatment a short time, his lungs measure *less* week after week, for then he is certainly dying by inches.

"But it does not follow, because a man does not measure to his full standard, that he is consumptive; it only shows the one thing—that he is defective as to the action and capacity of his lungs; that deficiency may be the result of decay, or debility, or from the lungs being crowded with phlegm or other fluids;. if the deficiency is not from decay, proper treatment will diminish that deficiency from week to week, because the treatment invites back the action of the lungs. Thus it is that the gradual *increase* in the capacity of the lungs to hold air, when that capacity, by any cause, has been diminished, is demonstrative of a return towards health.

"On the other hand, as persons are declining, the measurement decreases week by week, until there is scarce breath enough to enable them to cross the room, and soon they step into the grave.

